# Generating a Dynamic Synthetic Population – Using an Age-Structured Two-Sex Model for Household Dynamics

**DOI:** 10.1371/journal.pone.0094761

**Published:** 2014-04-14

**Authors:** Mohammad-Reza Namazi-Rad, Payam Mokhtarian, Pascal Perez

**Affiliations:** 1 SMART Infrastructure Facility, University of Wollongong, New South Wales, Australia; 2 National Institute for Applied Statistics Research Australia, University of Wollongong, New South Wales, Australia; University Toulouse 1 Capitole, France

## Abstract

Generating a reliable computer-simulated synthetic population is necessary for knowledge processing and decision-making analysis in agent-based systems in order to measure, interpret and describe each target area and the human activity patterns within it. In this paper, both synthetic reconstruction (SR) and combinatorial optimisation (CO) techniques are discussed for generating a reliable synthetic population for a certain geographic region (in Australia) using aggregated- and disaggregated-level information available for such an area. A CO algorithm using the quadratic function of population estimators is presented in this paper in order to generate a synthetic population while considering a two-fold nested structure for the individuals and households within the target areas. The baseline population in this study is generated from the confidentialised unit record files (CURFs) and 2006 Australian census tables. The dynamics of the created population is then projected over five years using a dynamic micro-simulation model for individual- and household-level demographic transitions. This projection is then compared with the 2011 Australian census. A prediction interval is provided for the population estimates obtained by the bootstrapping method, by which the variability structure of a predictor can be replicated in a bootstrap distribution.

## Introduction

Increasingly, planners require sophisticated insights into social behaviour and the interdependencies characterising urban systems. There is a growing need for new and evolving tools to assist research and decision-making. In order to create a realistic decision-making model, a reliable synthetic population of agents is required which should be able to reflect the social entities, individuals and households, with their characteristics and specific behavioural patterns. It is generally unrealistic to assume that all required information about the system could be provided by a single data source. Such a perfect single data source able to provide complete knowledge about agents' behaviour and their environment does not exist in practice. This paper presents an algorithm to generate a dynamic synthetic population for Wollongong, a region in the south Sydney Metropolitan area, from the multiple data sources.

The two main components in the population synthesizer are *initialization* and *evolution*. Initialization involves simulating the baseline area-specific population of individuals and households using sample-based unit-level data so that the simulated population meets aggregate-level information from the census. The result is a collection of simulated individuals grouped into five categories of households and associated with specific geographic locations. Data aggregation performed by initialization is uses a combinatorial optimisation (CO) method. Due to the individual-centered nature of the modelling framework, our implementation of CO is slightly different from traditional approaches described in the literature. In this paper, the influence of such modifications is investigated. After the baseline population is generated, we use a dynamic micro-simulation model for individual- and household-level demographic transitions over five years and provide a bootstrapping prediction interval given a significant level for uncertainty measurement. Our main objective remains to calibrate our algorithm against data from the 2006 Australian census, apply the five-year dynamic model and to validate the simulated population of 2011 against data from the 2011 Australian census.

The purpose of the reliable dynamic synthetic population is to create a valid representation of the population spatially distributed while addressing the daily population transitions. In this paper, we discuss standard approaches for generating a synthetic population. The relevant literature is reviewed in Section 2. Initialization of a synthetic population is discussed in Section 3. Population of households and individuals in 2006 living in Wollongong is generated using available information from the 2006 Australian census. An age-structured two-sex dynamic model is then presented considering the hierarchy within the population in Section 4. A five-year population dynamics within the target area is generated in this study and the simulated synthetic population in 2011 is validated against the 2011 Australian census (see Section 5). Discussion is provided in Section 6.

## Population Synthesis Methods

The synthetic reconstruction (SR) and hill climbing (HC) techniques are discussed in this section as two standard approaches for generating a synthetic population. The objective of the SR approach is to use a deterministic algorithm for reconstructing the population while the HC approach follows a stochastic data-driven procedure. In this section, these two approaches are compared and the advantages of the HC approach, the one which has been used in the current study, are highlighted.

### The SR Approach

Using the SR technique is a traditional way of generating a synthetic population based on both disaggregated- and aggregated-level data. This method first uses disaggregated-level data (which is usually sample data,) while assuming that it is a fully representative sample of the target population. This is generally referred to as the *seed data*. Then, in order to generate the synthetic population, individuals with the required socio-demographics are populated within each specific area using a weighting technique so that the marginal distribution follows the aggregated-level information coming from one source covering the complete population (which is usually census data). One way to do this is to use the deterministic re-weighting algorithm (e.g. [1] [2]) to allocate a certain weight to each unit record within the disaggregated-level data and consider the weights as a distribution of probabilities derived from the available aggregated-level data. Each attribute for the population units is treated separately and sampling from marginal distributions is conducted to select the number of units equal to the number of area-specific population totals. In practice, however, a perfect matching between all area-specific synthetic totals and the sample totals under the assumption that all of the areas are relatively homogeneous is unrealistic. An alternative way is to conduct a Monte Carlo sampling from the disaggregated-data based on the underlying conditional probabilities calculated as discussed by [3], rather than being deterministically re-weighted from the disaggregated-data. This is a stochastic approach as the conditional probabilities are readjusted in an iterative Monte Carlo sampling until a close match with the constraining tables or marginal distributions is achieved [4].

When the number of attributes required in the synthetic population increases, these approaches do not perform efficiently. In such cases, a popular approach is to estimate a joint distribution based on both disaggregated- and aggregated-level data. Then, the units are selected from the disaggregated-level data (which is usually a representative sample of the target population,) considering the joint probabilities estimated in the previous step. This technique is called the iterative proportional fitting (IPF) procedure and was first introduced by [5] who developed it for a non-geographical context. Relevant studies (e.g. [6], [7]) note that producing reliable multi-dimensional contingency tables for the IPF procedure needs a very representative sample. The theoretical underpinning of IPF procedure, as embedded in spatial interaction modelling was presented by [8]. The performance of the IPF procedure in generating disaggregated spatial data from aggregated data was evaluated by [9]. The research study by [10] was amongst the first attempts to use the IPF for activity-based transportation models.

Using the IPF procedure, only one level of hierarchy can be accounted for. For example, a synthetic population is generally required for different spatial micro-simulation purposes both at the individual- and household-level for which the IPF procedure is not an applicable method. In order to overcome this problem, [11] proposed a technique similar to the IPF procedure for generating the households while simultaneously improving the individual-level distributions. Another method, proposed by [12], was based on using the relation matrices to convert the distributions in order to convert the individual-level distributions to household-level distributions so that the marginal distributions could be controlled at both levels. Iterative proportional updating (IPU) procedure was then proposed by [13] to overcome this challenge. IPU is a practical heuristic approach which simultaneously controls the multiple hierarchy levels while adjusting the weights so that the distribution of individuals and households match as closely as possible [14], [15]. IPU adjusts the household weights based on the individual weights obtained from the IPF procedure by creating a new column for each combination [13]. This way, both individual and household levels converge, simultaneously. However, because IPU generates a new column for each element of the combination, it increases dramatically the memory requirements to run the model.

More recently, a research team from the Transportation Research Group at FUNDP-Namur (Belgium) facing heterogeneous information and levels of aggregation across different surveys decided to ‘construct individuals and households by drawing their characteristics or members at random within the relevant distribution at the most disaggregate level available, while maintaining known correlations as well as possible’ [16]. They proposed an algorithm consisting of 3 steps:

- A pool of available individuals is generated for a given area, namely the individuals‘ attribute joint-distribution denoted by *Ind*;
- The households' joint-distribution is estimated and stored in the contingency table *Hh*;
- The synthetic households are constructed by randomly drawing individuals from the individual's pool *Ind*, while preserving the distribution computed in the second step. Once a household has been built, it is added to the synthetic population.

### Hill Climbing (HC) Approach

Another family of synthetic population synthesizers is based on the HC approach. In computer science, HC is a relatively simple mathematical optimisation technique that covers a range of heuristics based on a random search. Considering many possible solutions for an optimisation problem, the HC technique starts with a random (potentially poor) solution and it generates an iterative process for maximizing an objective function, each time improving the result and finding a better solution. The HC algorithm terminates when the improvements in the iterative search process are negligible, which means a global optimum (or a hilltop) is reached with a negligible error [17]. The HC technique can be used for generating a synthetic population when populating a certain area with the units randomly picked from the seed data. If the simulated univariate distributions do not match the real area-specific marginal distributions, a certain number of simulated units will be selected and swapped with new units (from the seed data) so that the resulting population will be closer to the real one. This procedure will be repeated and the goodness-of-fit at each relocation step will be recorded as a measurement for possible improvement. The key aim is to reach a global optimum but sometimes the algorithm can be trapped in a local optimum or replacement can lead to deterioration. If we will be trapped in a local optimum or the deterioration will exceed a certain threshold, *random restart hill climbing* and *simulated annealing* are suggested to allow the algorithm to randomly restart at a more distant point, while keeping track of the global performance. This swap will help to avoid producing a poorer performance repeatedly [18]–[21].

An application of HC was designed by [22] based on the Combinatorial Optimisation (CO) method. This approach was first presented by [19], and it involves an iterative process. In order to generate the population for a certain area using the CO technique, a group of individuals is randomly picked from a disaggregate data set (e.g. a survey over a sample of the population over the target area) so that it matches the population size of the small area. The observed sample is then statistically compared with a pre-defined set of demographic characteristics of the target area. If the goodness of fit is not satisfactory, a record (either a household or an individual) in the observed sample is swapped randomly with one from the pool (i.e. the sample data). The statistical comparison is then carried out for the new observed sample. If this process enhances the overall performance of our desired combination, the replacement is made. This process is repeated with the aim of gradually improving the goodness of fit and is stopped when a desired accuracy in the comparison of statistics is met (which means that further improvements to the selected combination is negligible or impossible). Given the search space (the survey data), the final synthetic population from this approach is generally the best achievable at a given time, rather than a guaranteed optimal solution [21].

The first synthetic population generator adopting this approach was developed by the group led by Williamson [19]–[21]. Another major research effort to build a synthetic population using this approach was carried out at the National Centre for Social and Economic Modelling (NATSEM) based at the University of Canberra, Australia [23]–[25]. In this paper, an extended version of the combinatorial optimisation technique is used for generating a reliable synthetic population for the target geographic areas using available aggregated- and disaggregated-level information. The baseline population in this paper will be generated from the Confidentialised Unit Record Files (CURFs) and Australian census data. Then, this population needs to evolve over a simulated five-year period.

## Synthetic Baseline Population of the Wollongong Area

Generally, a perfect single data source which can provide complete knowledge about the population units does not exist. Therefore, researchers usually use multiple data sources to generate a more reliable and believable synthetic population. Typically, sample data is used to match the distribution of households and individuals and the census-based marginal distributions are considered in building the area-specific population. As an example, [13] used a sample-based IPU approach to generate the synthetic population of small geographies (blockgroups) in the Maricopa County of Arizona in the United States. Sample-free SR approach was used by [26] to generate a synthetic population of two municipalities in the Auvergne region in France. This approach was used by [15] to generate a synthetic population of Belgium at the municipality level. The aforementioned sample-free and sample-based approaches are compared by [27]. Assuming they had a population with complete data available about individuals and households, [27] compared a sample-free SR approach with a sample-based SR approach. They concluded that the selected sample-free SR method performed better for producing the population estimates because results using this method were globally closer to the real population values. While the level of uncertainty in the estimations and level of sensitivity of the algorithm to the number of variables of interest and their joint distributions are not discussed in this study, the conclusion may also apply for the IPU method in certain applications, especially where the access to a representative sample is very limited.

It should be noted that the sample-free methods (like what presented by [15],) must deal with the inconsistencies between margins extracted from the different cross-tabulations available. This takes time and energy to set the multidimensional weights for the population characteristics. In the sample-based method, these weights are calculated based on the sample data. In order to generate a realistic population for our target areas, a 1% Basic Census Sample File (CSF) available at CURFs is used as the seed data. This dataset contains the unit records of a 1 in 100 sample of occupied private dwellings along with the occupants of those dwellings (who spent the census night within Australia,) and a 1 in 100 sample of people from non-private dwellings along with the associated dwelling. It will be noted that CURFs contain the most detailed information available from the ABS. CURF microdata access is priced in accordance with the ABS Pricing Policy and Commonwealth Cost Recovery Guidelines available at the ABS website. Here, we use a CO technique for generating the baseline population of the target areas and apply an age-structured two-sex model for generating the population dynamics over a five-year period. Given a significant level for uncertainty measurement, we can then provide a bootstrapping prediction interval. As the uncertainty measurements depend on the variation originating from the seed data, having a representative sample is crucial to this study.

**Figure 1:**
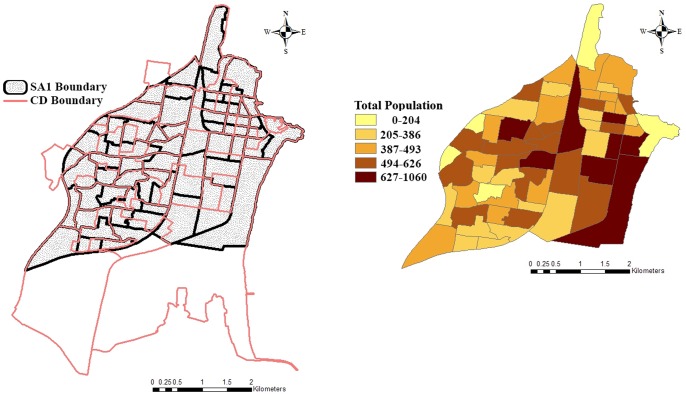
Map of the study area (SA2 Name: Wollongong) and SA1-specific total population in 2011.

From 1984 to 2011 the Australian Bureau of Statistics (ABS) used the Australian standard geographical classification (ASGC) for the collection and dissemination of geographically hierarchical classified statistics. In 2006, the smallest geographic area defined in the ASGC designed for use in the census of population and housing is the census collection district (CD) and serves as the basic building block in the ASGC. From July 2011, the Australian statistical geography standard (ASGS) has been progressively replacing the old ASGC. The ASGS was effective from 1 July 2011 as the new geographical framework and was utilised for release of data from the 2011 census of population and housing [28]. The smallest geographic area defined in the ASGS is the Mesh Block. Each Mesh Block has corresponding larger spatial boundaries of statistical areas level 1 (SA1), statistical area level 2 (SA2), statistical area level 3 (SA3), statistical area level 4 (SA4) and greater capital city statistical area (GCCSA). Further information about the transition from ASGC to the ASGS is available at the ABS website. The proto-population in this study is first generated based on the 2006 Australian census and therefore the proto-population is first generated per census district (CD) for the study area, and per statistical local area (SLA). Note that we can interconvert between SA1 and CD using the GIS information provided by ABS. A map of SA1s for the study area and the surrounding SA2: Wollongong is shown in Figure 1 while the boundaries of CDs are shown in a different colour. The SA1-specific total population sizes are also demonstrated in Figure 1.

The proto-population is first generated per Census District (CD) in the study area and per Statistical Local Area (SLA) for the target areas. We then need to assign individuals to households in such a way that we maintain a distribution as close as possible to both the individual demographics per CD or SLA but also the household demographics of that area. Since the Australian Census is a complete enumeration of local residents and visitors on the night of the census, we can use closed-form solutions to our set of constraints in order to find the closest match between our proto-population and the actual population demographics per CD both at the individual and household levels. Once the baseline population is generated for different CDs, we interconvert between SA1 and CD using the GIS information provided by the ABS and generate the SA1-specific population. This enables us to compare the simulation-based estimation results with the 2011 census data.

### Hierarchical Structure for the Synthetic Population

**Figure 2:**
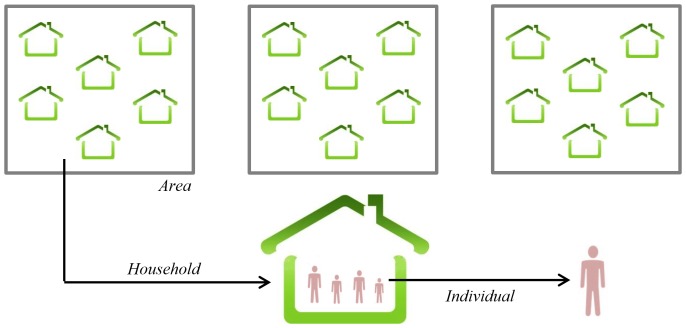
Two-fold nested structure of the individuals and households within the target areas.

This paper addresses the structural hierarchies in designing a synthetic population in which household structures and the socio-demographics of individuals living within the household are considered. Here, *G* denotes the total number of geographic areas in our study and *N_g_* denotes the total number of individuals located at the *g* ^th^ area while *M_g_* is the total number of households located at such area. Single-member and multi-member households within the population are considered, each household with different socio-demographic characteristics. In order to address the hierarchies within the population while distinguishing between this structure and cross-classification, we will refer to it as a two-fold nested design (see Figure 2). This technique has been used by researchers such as [29]–[31]. A certain number of characteristics (denoted by *P*) are considered in this study to define any specific individual within the synthetic population. The individuals within the synthetic population will then form single-member and multi-member households with certain demographics. This is while a certain number of characteristics (denoted by *Q*) is considered for each household. Given a finite set *P* of individuals and a finite set *Q* of households, the *state model* here is defined as an onto (surjective) function from *P* to *Q* (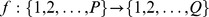) satisfying certain additional constraints, where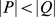.

**Figure 3:**
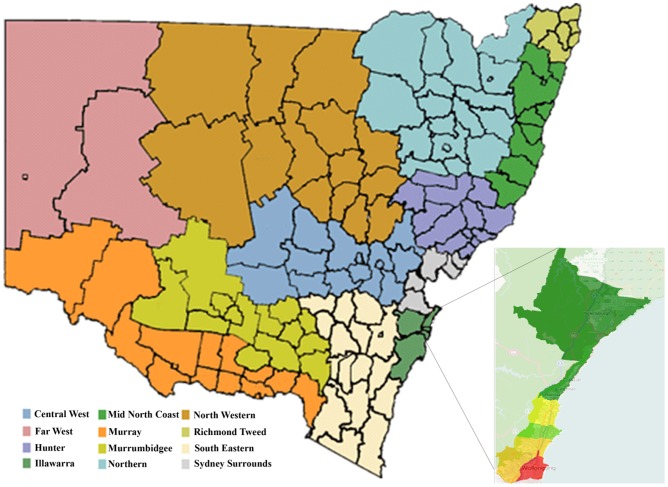
NSW map clustered by geographical regions and a selected study area (SA3 Name: Wollongong) (Helensburgh, Thirroul, Austinmer, Coalcliff, Woonona, Bulli, Russell Vale, Bellambi, Corrimal, Tarrawanna, Balgownie, Fairy Meadow, Figtree, Keiraville, & Wollongong).

In order to generate a realistic population for our target areas, 1% CSF is used as the seed data. The proto-population in our study consists of individuals defined by their respective age, sex and household type informed by the 1% CSF based on the 2006 Australian census while considering the area-specific census tables. The 1% CSF is available at a certain geographical level. The geographical regions for which the 1% CSF is given for New South Wales (NSW) and are presented in Figure 3. As can be seen in Figure 3, the area SA3:Wollongong is located within the Illawarra region and our study area (SA2:Wollongong) is highlighted in red on the south-east of SA3:Wollongong. In order to generate the baseline population, it is assumed that the population distribution for the target area will be the same as the population distribution within the Illawarra region. This enables us to use the 1% CSF and the 2006 census tables in order to generate the proto-population at individual and household levels using the combinatorial optimisation method discussed in Section 2.

Using the 1% CSF and the household-specific information available for the target area, the baseline population is generated. The result is a collection of simulated individuals grouped into 5 categories of households to be consistent with the Australian census categorization. Household attributes considered in this study are (*i*) couple only, (*ii*) couple with children, (*iii*) lone person, (*iv*) one parent and (*v*) other family. The objective of this study is to generate a synthetic population from the data provided by the ABS. To be realistic, the baseline population should adequately match the distribution of individuals and households living in a given area as per the demographics information provided by the Australian census. The population involves a merging of individual and household level datasets. The merged datasets have to be a valid representation of the original datasets and preserve data validity.

### Combinatorial Optimisation Algorithm

One issue that is relevant to our case but is not addressed in all aforementioned studies which adopted the SR and CO approaches is the constraints of the composition of residents in households by individual types. The traditional CO and SR approaches can generate a synthetic population that satisfies the above constraints if categories of households and individual relationships in the survey data match with those in the census data. While this may not often be the case in practice, one can overcome the issue of category mis-match by re-categorizing households and individual relationships in the survey data. However, the level of accuracy loss as a result of such a re-categorizing process is unknown. The accuracy is also dependent on the size of the survey data, as mentioned in the previous section. The SR approach also requires additional cross-tabs, for example the number of people by relationship category by household category, as it is likely that the census data does not provide a full joint distribution of all desired characteristics. A serious problem with generating these cross-tabs in our study using the IPF method and/or its variations is data inconsistency. The Australian Bureau of Statistics (ABS) introduced small random adjustments to tables of census data so they can be released without breaching confidentiality. Corrections to these tables may be needed to ensure marginal distributions of the desired characteristics are consistent before any iterative processes of proportional fitting are carried out to ensure convergence of these processes. Also, the resulting cross-tabs may not satisfy the above constraints of resident composition by individual types.

We propose a methodology to generate a synthetic population for small areas in the target region, (CDs,) based on the aggregate census data of each area and knowing that that disaggregate data of household and individual records is partly available from the 1% CSF. Attributes of individuals in the synthetic population are age, gender and household relationship. Attributes of households are household category, classified by the composition of relationship of residents. The objective is to ensure the final synthetic population satisfies the demographic statistics not only at individual level but also at household level, including the constraints of the composition of residents in households by their relationships. Because of the random adjustments made to the ABS tables, the methodology also presents a framework to identify and remove outliers in these tables before records of individuals and households are instantiated.

Assuming the population characteristics within the sub-regions of Illawarra to be distributed homogeneously, we pick a sample using simple random sampling without replacement, from the seed data, which is the 1% Basic CSF available for the whole Illawarra region. Then, we compare the marginal distribution of this sample with CD-specific information available in the 2006 census tables. The resulting marginal distribution should match the actual information available in the 2006 census tables. Using the CO techniques discussed above, a certain number of population units are then swapped with population units from the seed data in order to get closer to the actual marginal distributions. This process is repeated until an optimum sample is obtained in terms of quadratic function of the estimators calculated using the sampled data, which is:



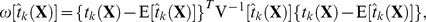
where 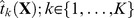 is an estimation for *k* ^th^ population characteristic out of the matrix of sampled units denoted by **X**. Here, 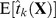
is the expected value for 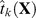, while 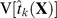 is the variance of 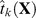. Note that in this study, the total number of different types of individuals and households is to be estimated.

Here, the baseline population is generated for the target areas based on the 1% CSF generated from the 2006 Australian census while the marginal distributions provided by the 2006 census (age, sex and household type) for each CD are considered. The proto-population consists of individuals defined by their respective age, sex and the type of household. We then need to assign individuals to households so as to maintain a distribution as close as possible to both the individual demographics per CD or SLA and also the household demographics of that area. The CO approach is used, but when the marginal distributions for a certain CD given by the ABS are not reached, some units are randomly swapped with sample units available from a larger area (such as the SLA). This iterative process will continue until the goodness-of-fit reaches a certain point. Then, we allocate each household to a street block in the target CD.

The sets of ABS data used for this purpose include individual-related tables (e.g. distribution of age by gender, and the relationship in household by age and by gender) as well as household-related tables (e.g. family composition, and family composition by gender of persons in family). The core of the algorithm aims at merging individual- and household-level data sets, which is centered on the correction of any discrepancies in common attributes among datasets (as random errors are introduced to values in ABS tables to avoid the release of confidential data). When the proto-population is allocated to CDs, we need to generate the dynamics of our population caused by naturally occurring events such as birth, death, marriage, divorce and relocation. To do this, it is crucial to develop an age-structured two-sex population model that explicitly addresses the issue of matching females and males into couples. This model is crucial in settings where the traits of children are determined by the traits of both the mother and father. Age-dependant changes in socio-demographic situations and social convergences should be studied in order to generate a more robust synthetic dynamic model.

## Modelling the Population Dynamics & Household Projections

The early attempts at modeling population dynamics using mathematical formulas date back to 1662 when John Graunt presented the first examples of life tables using the parish records of birth and death in order to describe the demography of London [32]. The basis for most current modeling of dynamics for different biological populations is the mathematical model of population growth discussed by Englishman Thomas R. Malthus (1766–1834). He tried to model population growth using a fixed proportion increase over a given period of time regardless of the initial population size. This model, the Malthusian growth model, was published in 1798 in the essay entitled ‘An essay on the principle of population’. Then, Benjamin Gompertz and Pierre François Verhulst refined and adjusted the Malthusian demographic model [33]. In the last two decades, microsimulation models have played an increasingly key role in picturing the population dynamics (e.g. [34]) and forming government policies (e.g. [35], [36]) in many countries such as Belgium [37], Norway [38], Sweden [39], [40], the United States [41]–[43], the United Kingdom [44], [45], Canada [46], [47], and Australia [23], [48]. Following the scientific literature, in this section we present a dynamic microsimulation model for individual- and household-level demographic transitions.

### Individual Dynamics

The general method for estimating density-independent population growth is to assume that the population grows at the same rate ‘*1+R*’ regardless of the population size. Then, the total population at year *t* can be mathematically calculated using the Malthusian population model as below:



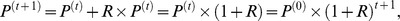
where *P* ^(*t*)^ and *P* ^(*t*+1)^ denote the population size during year *t* and *t*+1, respectively and *R* denotes the population growth rate per year (the so-called Malthusian parameter [49]). Here, the population size at the starting year (of a simulation) is assumed to be known and is denoted by *P* ^(0)^.This model simply shows exponential growth in the population and is often referred to as ‘The exponential law’ in the field of population ecology and is regarded as the first principle of population dynamics.



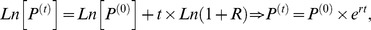
 where, 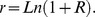



In order to generate a general form of the density-dependent population growth model, the population growth rate is assumed to change over the years and is defined as a function of time. Often the population growth at each year in such models is defined as a function of population total at that time period [50], [51]. This model is generally used in cell biology as a negative density-dependence or density-dependent restriction considering the population growth to be curtailed by crowding, predators and competition. A general form of mathematical model based on biological theory is presented in the literature (e.g. [52], [53]) as follows:
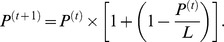
where parameter *L* denotes the carrying capacity of the environment and represents the maximum individuals in the system according to the defined conditions. The population growth will be positive using the above model when 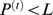, while the population will decrease when 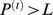. The population growth will be equal to zero when 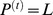. These models and their generalization incorporating age-structures like the one presented by [54] are restricted to modeling the dynamics of a homogenously mixing population and do not take into account the gender-based factors such as mating which are crucial when studying the life history of a real population [55].

The consistency problem in modeling the population dynamics considering the pair information was noticed by [56] who looked at the net reproduction rates in France for males and females separately from 1920–1923. Deterministic and stochastic models using the pair formation process were then presented by [57] and [58]. Then, [59]–[63] focused on the difficulty in handling the genuine sex parameter in population dynamics and the interactions with other population behaviours. The two-sex population models with vital processes independent of age do not consider the age structure and this may cause some bias in predicting the reality. Although this sort of model seems to be well understood by mathematical demographers, much remains unknown about age and sex structure models. To deal with such problems, [64] proposed a model with no age structure and developed a quasi-age structure model while [65] presented an age-structured two-sex model in which couples dissolve only through the death of a spouse and widows never remarry. Many researchers such as [66]–[73] worked with two-sex population models both with and without age structure. The impact of demographic ageing within the human population was evaluated using the modeling techniques proposed by [55], and [74].

Here, we assume discrete time sections to facilitate the required calculations. Like [75], we divide each year into 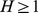 periods of length 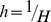. It is also assumed that all individuals will die before reaching *w* years, so the age set is: 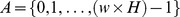.

In the current study we assume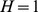. Assuming the birth rate and death rate to be known for males and females at different ages, the population growth from time ‘*t*’ toward time ‘*t*+1’ is calculated using the model presented below:



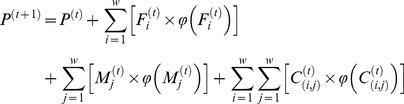
where, the number of males of age *j* at year ‘*t*’ is denoted by 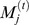 while the number females of age *i* on this year is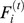. Here, 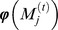 and 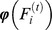 are the population growth rates for the male and female respectively, of a certain age at year ‘*t*’. The rate of population growth for the male or female of a certain age in this study is equal to the immigration rate minus emigration rate minus the death rate. The element of natural birth is considered in this model using the total number of couples with a female individual of age *i* and a male of individual of age *j* denoted by 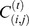, and the birth rate considered for these couples is denoted by 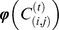.

As discussed, we require some information about the existing couples within the population at each year. The age-structured model for presenting the couple population is as follows:
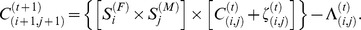
where 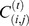 denotes the existing number of couples at time ‘*t*’ with females of age *i* and males of age *j*, while 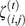 is the number of new couples during year ‘*t*’. Number of divorces at year ‘*t*’ with females of age *i* and males of age *j* is denoted by 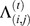. Then, 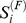 and 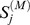
are the survival rates for females of age *i* and males of age *j*, respectively. The probability of being widowed is considered in our model as: 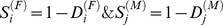.

Note that, 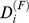 is the death rate for the females of age ‘*i*’ while 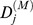 is the death rate for the males of age ‘*j*’. Note that, the same sex- and age-specific death rates are considered for married and single individuals in this paper. Here, we present two models which calculate male and female widow/widower population at time ‘*t*+1’. The model for the widowed females is as follows: 
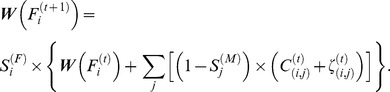
where 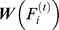 and 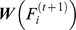 repectively denote the total number of widows at year ‘*t*’ and ‘*t+1*’ while the subscript *i* shows the age of the widows. Using the formula presented above, the total number of widows at year ‘*t+*1’ is equal to the total widows at year ‘*t*’ plus the number of new widows caused by the death of males in all existing couples. The number of female who may die during this year is also considered by adding the term 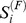 which is the survival rate for females with of age *i*. Using the same technique, we can define a model for the widowers in year ‘*t*+1’ as follows:



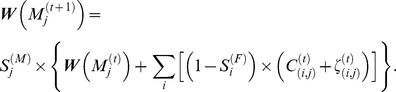
where 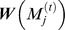
and 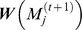
denote the total number of widowers at year ‘*t*’ and ‘*t+*1’, respectively, while the subscript ‘*j*’ shows the age of the widowers.

### Household Transitions

Projecting the dynamics of family formations and behaviors (family size, family structure and family life course) across time and place depends on compositional changes in the population's socio-demographic structure. A computer-simulated synthetic population for Australia was discussed by [76]. For the evolution of this population, he used an extended version of the dynamic household model proposed by [77]. However, they have modeled the population dynamics for the whole of Australia and they did not consider the diversity of the population characteristics in smaller areas. A method presented by [78] generates the initial population without a sample. The main objective of this research is to present a more comprehensive model for producing household projections in the Wollongong area across time, based on the four vital rate components: fertility, mortality, marriage and divorce. For young single individuals, we also consider the probability of leaving the parental home and establishing their independence. We first generate a synthetic population from the census data provided by the Australia Bureau of Statistics. Then, we use our model to project the household structures within the population over time.

The focus of this research is to present a reliable method for producing household projections over time. While the model needs to be relatively simple, with modest data requirements and low computational intensity, it must be able to generate realistic links between demographic events and changes in household structures [79]. Here, we present the probability function of household formation and dissolution. First, we suppose that we have *H* Household Types (HHT*s*). The main goal is to define the transition function of household types from time *t* to time *t*+1. Here, we assume the main events that determine new household formations at time *t*+1 are: *i*)death, *ii*)marriage, *iii*)birth, *iv*)divorce and *v*)leaving the parental home. As we are considering different structures for the households (depending on the demographic characteristics defined for the people living in that household) at time *t*, we must consider the transitional rates of the five major events during a time period so as to cover almost every possibility. The probabilities for occurrence of all possible events considered in our study are:

- Death rate: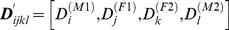,
- Marriage rate:
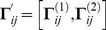
,
- Birth rate: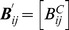,
- Divorce rate: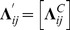, and
- Leaving the parental home: 
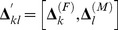
.

Here, the death rates for the independent individuals (singles or couples) and the individuals living with their parents have been considered separately. Here, 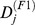 and 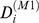
denote the death rates for independent female individuals of age *j* and independent male individuals of age *i*, respectively. If there is an existing dependent individual (child) living in a household, the death rate for such an individual is considered seaparately. The death rate for dependent female individuals of age *k* is denoted by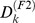 while the death rate for dependant male individuals of age *l* is denoted by 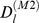. The vector 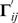 includes the marriage rates considered in our study. In our study, matrices are shown in bold. Here, we consider the rate of first marriages between female individuals of age *i* and male individuals of age *j* (denoted by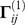). When one of these individuals has been married before, a different notation is used for the new marriage (denoted by 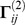). Here, we assume that the birth rate for a selected family can be calculated based on the age of parents. This rate is denoted for a married male and female of age *i* and *j* as 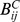. The divorce rate for such a couple is 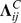. For dependant individuals over 15, we consider the rate of leaving the parental household using the vector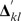. Here we assume that the each household may have one female or male dependant individual over 15 who may decide to leave their family. This model can be easily extended to more complicated cases by adding new elements to the matrices presented above.

In respect to transition of the household type *g*_1_ at time *t+*1 (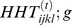), to the household type 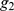
at time *t+*1 (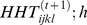) (where 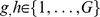, and 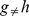), we can define an 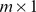 dimension indicator vector denoted by 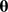 as the vector of key components or demographic parameters of the transition function. Generally, the dimension of this vector is 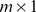
where *m* is the number of key components mentioned above, and here *m* is ten. Note that, *g* and *h* denote two different states of households, including the ages of their members while indexes *i*, *j*, *k,* and *l* refer to the age of male parent (or adult), female parent (or adult), female children, and male children within the household, respectively. In a household transition, certain events have happened. Here, 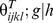 is a vector of 0s and 1s. Where an event (or a component) affects the household transition, the associated element in this vector is equal to 1. Otherwise it is equal to 0. The vector 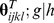 is defined by:
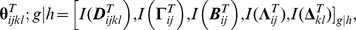
where
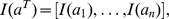
and
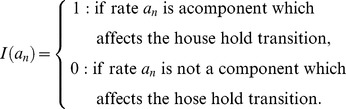



Consequently, we define the 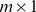 probability transition vector based on the effective components mentioned above, as:
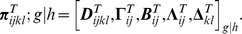



Using the above indicator and probability transition vectors and assuming the independent events, we can obtain the overall probability of moving 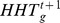 from 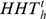, 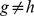. It is
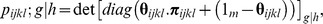
where 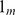 is the 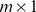
vector where all elements are 1. If the transition 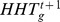 from 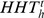, 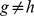, is not unique and there are *D* out of 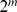 possible cases to make the transition of interest. Using the inclusion-exclusion principle in the probability addition rule [80], the overall probability can be written by:
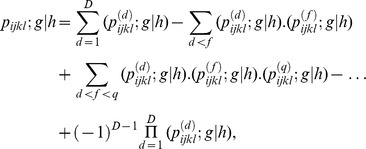
which can be written in closed form as:
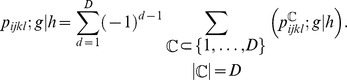



Now the overall probability of moving 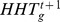 from 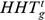 is given by:
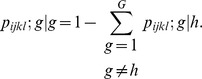



Using the above probabilities, we can produce the *HHT* transition probability matrix.

## Results: Simulating the Dynamic Synthetic Population of the Wollongong Area

According to the ABS values from the 2006 census for the population living in private dwellings in the study area (SA2: Wollongong), there are approximately 25890 individuals and 16870 households. The 2011 Australian census shows that 27076 individuals were living in the study area on the night of the 2011 census. In this paper, an immigrant population is added to the existing generated population at the end of each simulation step (i.e. a year). The demographic characteristics of the simulated immigrant population are assumed to be consistent with the marginal distribution considered for the demographic characteristics of the current population in each of the five household categories.

### Generating the Proto-Population

As mentioned in Section 2, the baseline synthetic population in this paper is generated based on 2006 Australian census data and should be simulated at the CD levels as the 2006 census data is available for this level. Then, the concordance between SA1s and CDs (presented in Figure 1,) is used to allocate the baseline population to the SA1s. The population dynamics will then be applied to this population. This way the population estimates in five years can be compared with the 2011 Australian Census data which is available at the SA1 level.

**Figure 4:**
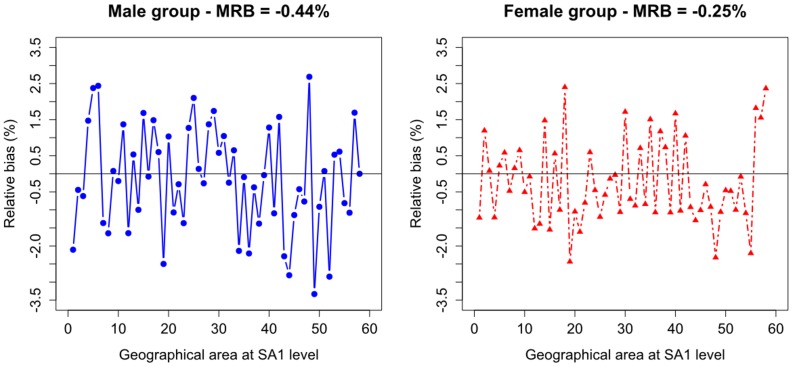
Relative bias of SA1-specific total male and female estimates in 2006.

Here, we present the relative bias for selected 2006 population estimates. Relative bias (RB) is a measure in percentage which magnifies the difference between actual quantity and the estimate. Suppose a sample 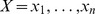 is used to estimate a quantity of interest 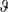 and let 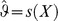 be a statistic that then estimates the RB of 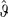 using: 
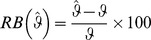



If there are 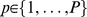 unknown parameters 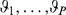 then to simplifying the overall biasMedian relative bias (MRB) is as follows:
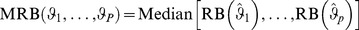



The two graphs presented in Figure 4 demonstrate the RB of estimated male and female totals in 2006 for all SA1s in our study area. As shown, the RB presented in Figure 4 is stationary, randomly distributed around zero. The MRB of the SA1-specific total male and female estimates in 2006 are respectively equal to −0.44% and −0.25%, respectively. Therefore, we can conclude that our proto-population is not far from the reality.

**Figure 5:**
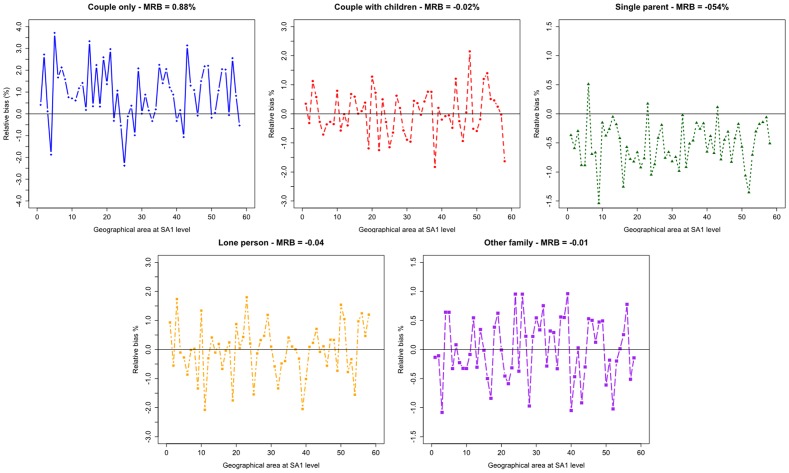
Relative bias of SA1-specific estimates for total number of different households in 2006.

The RBs of estimated totals for different households considering the five categories of HHTs are presented in Figure 5. The results show that the total households generated within the SA1s are distributed similarly to the 2006 Census data. As can be seen, the total SA1-specific single parents are mostly underestimated with the MBR equal to -0.54%, which is negligible. Note that the SA1-specific totals are estimated with higher RB for SA1s with smaller populations.

### Population Dynamics in the Study Area

In order to generate the dynamics within the synthetic population simulated for our target areas, vital statistics of death, marriage, birth, divorce and leaving the parental home are required. Unfortunately, these rates are not available to this study for the target areas (CDs and SLAs). Therefore, the rates available for the larger areas are used and possible changes in these rates over time are ignored. The main purpose in this section is to take the baseline population generated for 2006 (based on the 2006 Australian Census,) and evolve the population over five years. The results can be compared with available information from the 2011 Australian Census and this can be used to evaluate the simulation approached proposed in this paper.

**Figure 6:**
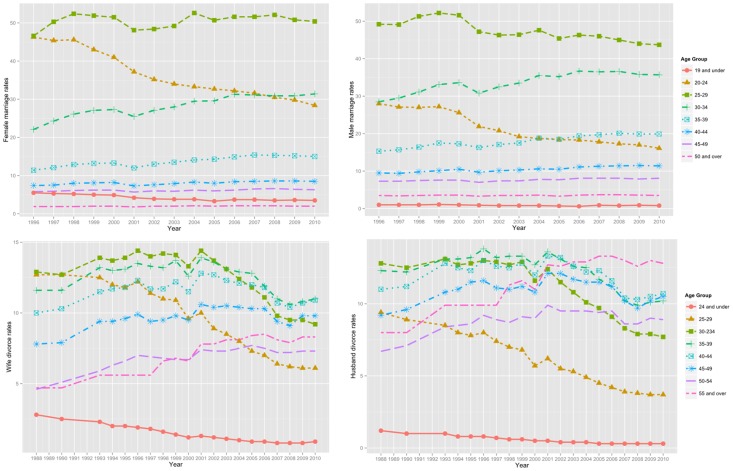
Two-sex Age-Specific Marriage & Divorce Rates in NSW 1987–2010.

The age-specific birth and death rates are available at the state level in Australia and are used in the current study. These rates for New South Wales (NSW) are presented in Table 1–2. Age- and sex-specific marriage and divorce rates in NSW are presented over time in Figure 6 (source: ABS; 3301.0 BIRTHS, Australia, 2010 & ABS; 3302.0 DEATHS Australia, 2010). While the target rates have been changed from 1996 to 2010 for different age groups, the changes from 2008 to 2010 are almost negligible.

**Table 1:** Age-specific Birth Rates Per 1000 for in NSW 2010.

Age	Females
**15–19**	15.3
**20–24**	51.4
**25–29**	101
**30–34**	120.4
**35–39**	63.4
**40–44**	11.3
**45–49**	0.6

**Table 2:** Age-specific Death Rates Per 10000 for in NSW 2010.

Age	Gender	
	Males	Females
**0–4 years**	64.1	63.3
**5–14**	24.9	16.8
**15–24**	133.7	43.1
**25–34**	242.9	122.6
**35–44**	507.5	268.3
**45–54**	1093.5	575.5
**55–64**	2176.8	1445.8
**65+**	8960	8008.4

### 2011 Population Estimates and Uncertainty Measurements

In this paper, the baseline population was generated for the study area using 2006 Australian Census. Then, the area-specific population attributes were predicted for 2011. Any inference about uncertainty in prediction is to be based on a complex procedure for which theoretical results are unavailable or not useful for the sample sizes met in practice. Bootstrap methods based on resampling techniques can be an alternative for analytical procedures when the distribution of population estimates is unknown [81]–[83]. The basic idea of the bootstrapping method is that, in the absence of any other information about the distribution, the observed sample contains all the available information about the underlying distribution, and hence resampling the sample is the best guide to what can be expected from resampling from the distribution.

The basic principle underlying the bootstrap method in various settings is sample set. The bootstrap technique attempts to recreate the relation between the ‘population’ and the ‘sample’ by considering the sample as an epitome of the underlying population. Generally, this reflection can be obtained properly by resampling from the sample set to generate the ‘bootstrap sample’, which serves as an analog of the given sample. If the resampling mechanism is chosen appropriately, then the resample, together with the available sample, is expected to reflect the original relation between the population and the sample. The advantage derived from this exercise is that, in practice, it is possible to avoid the problem of having to deal with the unknown population directly, and instead, use the sample and the resamples, which are either known or have known distributions, to address questions of statistical inference regarding the unknown population quantities. The bootstrap principle is most transparent in the case where random variables are independent and identically distributed (iid) [84]–[86].

Suppose that sample 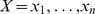 is used to estimate a distribution parameter 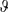 and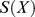 to denote the estimate of such a parameter (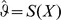). For the purpose of statistical inference of 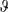, we are interested in sampling the distribution of 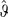. This way we can calculate the accuracy of the estimator obtained or set the confidence intervals for that. In many applications, however, the sampling distribution of 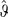 is intractable. If the true distribution 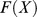 were known, we could draw samples 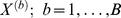 from 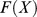 and use Monte Carlo methods to estimate the sampling distribution of the estimate 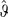. Since 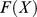 is unknown and we cannot sample from it, the bootstrapping idea suggests resampling the original sample instead. The distribution from which the bootstrap samples are drawn is the empirical distribution. Let sample 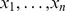 of independent real-valued random variables follow probability distribution function 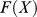. Then,



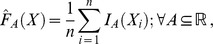
where *A* is the sample space, 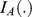 is an indicator function with values 1 and 0, and 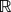 is set of real numbers. Then, a bootstrap estimate of uncertainty (standard error) can be obtained by the following algorithm:

1. Drawing *B* independent bootstrap samples with replacement from set 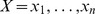


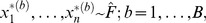

2. Evaluating the bootstrap replications 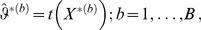

3. Estimating the standard error 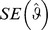 by the standard deviation of the *B* remplications 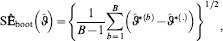

where
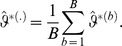



The bootstrap technique enables us to derive estimates of standard errors and confidence intervals for complex estimators of complex parameters of the distribution and to assign measures of accuracy to the sample estimates and in practice the simplicity of this method is a great advantage. While confidence intervals are extremely valuable in statistical inference, bootstrap confidence intervals are asymptotically more accurate than the standard intervals obtained using sample variance and assumptions of normality [87]. Recently, bootstrap method is used for measuring the uncertainty of the micro-simulation and activity-based models [88]–[90]. Here, the bootstrap confidence intervals are calculated for the estimated area-specific totals using the dynamic micro-simulation model for individual- and household-level demographic transitions. While performing replications of the simulations to obtain the required uncertainty measurements is computationally more expensive than the bootstrapping method, bootstrapping is also an appropriate way to control and check the stability of the results. Here, we use the bootstrap procedure presented previously for calculating the uncertainty for the resulting population estimates based on ‘*B = *1000’ independent bootstrap samples out of the initial population and running the whole micro-simulation based on each sample, separately.

1. Given an optimized sample set 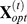 at time *t* we draw a simple random sample with replacement from the set 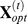. This bootstrap sample is denoted by 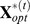.
2. Using 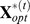 we calculate the population characteristics at time *t*+1 denoted by 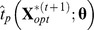. For simplicity, we can write this bootstrap prediction as 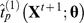 where superscript (1) stands for the first bootstrap replication.
3. We repeat these steps *B* times to obtain *B* sets of predictors for the *p* ^th^ characteristic as follows:
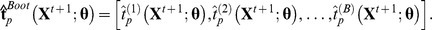

To obtain the uncertainty measurement for 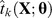 we use a 95% nominal bootstrap confidence interval. This interval is:



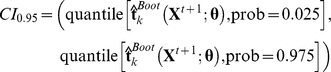



**Figure 7:**
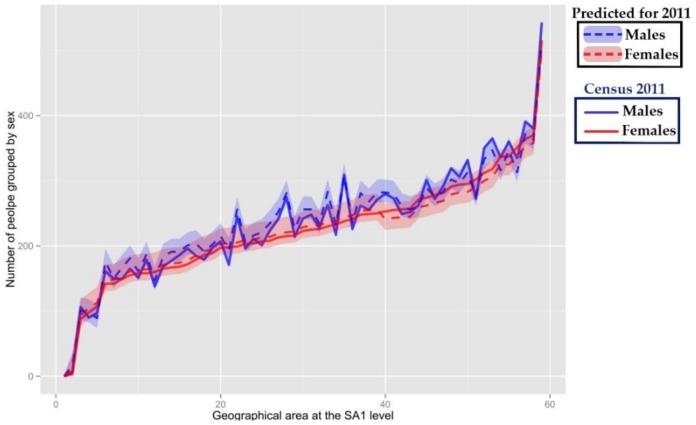
Age-specific population predictions vs the 2011 Australian Census at SA1 level.

Figure 7 demonstrates the number of males and females predicted for each SA1 within the study area. Note that the graph illustrates the age-specific population predictions and is generated so as to so as to be ascending for the area-specific total females.

**Figure 8:**
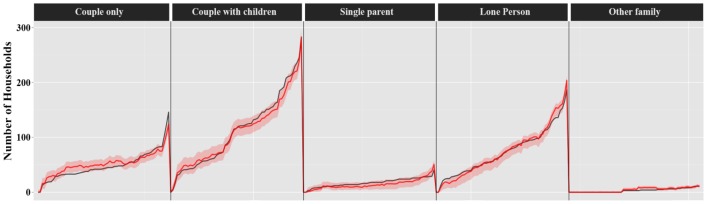
Household type-specific population predictions vs the 2011 Australian Census at SA1 level (red line: predicted values; black line: the actual numbers based on the 2011 census data; area in light red: 95% nominal bootstrap confidence interval calculated for the predicted values).

Figure 8 demonstrates the ascending graphs for the predicted number of different households in 2011calculated in this study for SA1s in Wollongong. The red line shows the predicted values while the black line demonstrates the actual numbers based on the 2011 census data. Although there are some differences between the estimated and actual numbers (of males, females and different household types), the actual values fall within the 95% nominal bootstrap confidence interval calculated for the predicted values for almost all SA1s.

**Figure 9:**
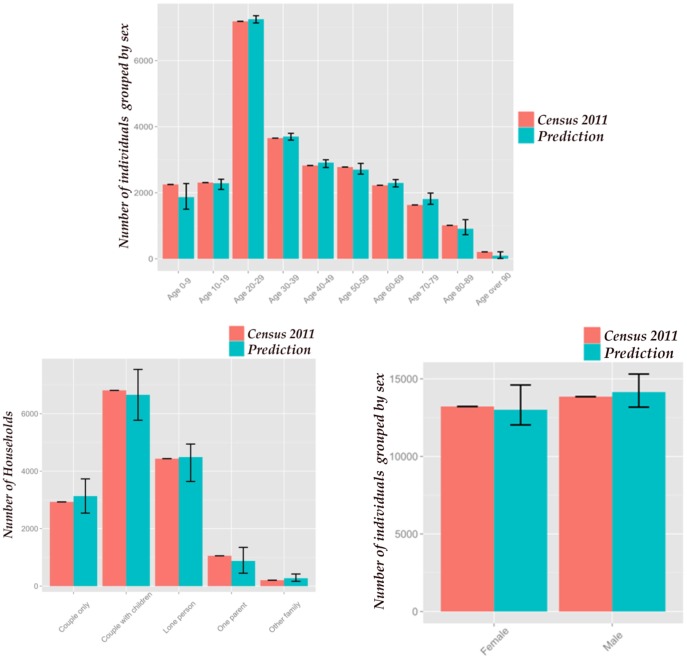
Population predictions vs the 2011 Australian Census for the whole study area.

The aggregated-level information predicted for the whole study area is compared with the 2011 Australian Census using the graphs presented in Figure 9. The estimated number of people within different demographic groups is a reasonable match with the actual values based on the 2011 census. However, some estimates such as the number of individuals between the ages of 0–9 and 70–79 and the number of person-only and parent-only households have larger errors. This might be due to possible differences between the state-level vital rates available to this study for population evolution and the real value of these rates.

## Discussion

Families are changing in many ways across time and projecting the dynamics of family formation behaviours (family size, family structure and family life course) is useful for planners to come up with solid decisions for improving the quality of public social services and urban management. Our aim in this paper was to study the effect of inter-individual interaction in order to model individual behaviours while considering available description of life-course dynamics, realistically [91]–[93]. Household forecasts such as changes in the number, size and composition of households, are crucial for planning the housing supply [94]–[97], household savings and consumption patterns [98]–[102], and environmental consequences and urban household energy transition [103]–[112]. A dynamic synthetic population is generated in this study for a certain region of NSW with given assumptions considering household transitions and the results are validated against the 2011 census data. The results show that the synthetic population generated for 2011 is not far from the real population.

The main purpose in this paper was to generate a reliable dynamic synthetic population of the study area based on available data. Evolution discussed in this paper involves the ageing of each individual and drawing of age-dependent life-event probabilities (birth, death, marriage, divorce and leaving the parental home). Unfortunately, these vital rates are not available to this study for the target areas (CDs and SLAs) and the rates available for the larger areas are used and possible changes in these rates over time are ignored. While we expect the algorithm to be sensitive to these rates in generating the dynamic synthetic population, the results show that we are not far from the reality. This means that the vital rates for our study areas are not very different to the larger areas. The occurrence of the life events influences both individual and household entities. Evolution uses a discrete event simulation model with a time-dependent feedback loop triggering either probabilistic or incremental changes to individual states. At any point in time, each individual is characterized by a significant number of state variables. These state variables describe personal attributes such as sex and age, relationship to a, i.e. family status and is based on particular geographic locations. This means that the current model does not take into account social networks or the ability for agents to learn from each other. While we recognize these are major features of real life, corresponding enhancements to the model will need to be addressed later on in future research studies. It is also noted that the required rates for the changes within the population are not available for the target small areas (CDs and SA1s). Therefore, the rates available for a larger area are used and possible changes in these rates over time are ignored due to the limited availability of data.
