# Correction: Generating a Dynamic Synthetic Population – Using an Age-Structured Two-Sex Model for Household Dynamics

**DOI:** 10.1371/journal.pone.0104138

**Published:** 2014-07-25

**Authors:** The PLOS ONE Staff

The first author’s name is incorrect in the citation. The correct citation is:

Namazi-Rad M, Mokhtarian P, Perez P (2014) Generating a Dynamic Synthetic Population – Using an Age-Structured Two-Sex Model for Household Dynamics. PLoS ONE 9(4): e94761. doi:10.1371/journal.pone.0094761

The first subheading in the Population Synthesis Methods section is incorrect. The correct subheading is:

Synthetic Reconstruction (SR) Approach.
